# Development of performance and learning rate evaluation models in robot-assisted surgery using electroencephalography and eye-tracking

**DOI:** 10.1038/s41539-024-00216-y

**Published:** 2024-01-20

**Authors:** Somayeh B. Shafiei, Saeed Shadpour, Farzan Sasangohar, James L. Mohler, Kristopher Attwood, Zhe Jing

**Affiliations:** 1 Intelligent Cancer Care Laboratory, Department of Urology, Roswell Park Comprehensive Cancer Center, Buffalo, NY 14263 USA; 2 Department of Animal Biosciences, University of Guelph, Guelph, Ontario, N1G 2W1 Canada; 3 Department of Industrial and Systems Engineering, Texas A&M University, College Station, TX 77843 USA; 4 Department of Urology, Roswell Park Comprehensive Cancer Center, Buffalo, NY 14263 USA; 5 Department of Biostatistics and Bioinformatics, Roswell Park Comprehensive Cancer Center, Buffalo, NY 14263 USA

**Keywords:** Learning and memory, Visual system, Education

## Abstract

The existing performance evaluation methods in robot-assisted surgery (RAS) are mainly subjective, costly, and affected by shortcomings such as the inconsistency of results and dependency on the raters’ opinions. The aim of this study was to develop models for an objective evaluation of performance and rate of learning RAS skills while practicing surgical simulator tasks. The electroencephalogram (EEG) and eye-tracking data were recorded from 26 subjects while performing Tubes, Suture Sponge, and Dots and Needles tasks. Performance scores were generated by the simulator program. The functional brain networks were extracted using EEG data and coherence analysis. Then these networks, along with community detection analysis, facilitated the extraction of average search information and average temporal flexibility features at 21 Brodmann areas (BA) and four band frequencies. Twelve eye-tracking features were extracted and used to develop linear random intercept models for performance evaluation and multivariate linear regression models for the evaluation of the learning rate. Results showed that subject-wise standardization of features improved the R^2^ of the models. Average pupil diameter and rate of saccade were associated with performance in the Tubes task (multivariate analysis; *p*-value = 0.01 and *p*-value = 0.04, respectively). Entropy of pupil diameter was associated with performance in Dots and Needles task (multivariate analysis; *p*-value = 0.01). Average temporal flexibility and search information in several BAs and band frequencies were associated with performance and rate of learning. The models may be used to objectify performance and learning rate evaluation in RAS once validated with a broader sample size and tasks.

## Introduction

The benefits of robot-assisted surgery (RAS), and more specifically, the da Vinci Surgical System (Intuitive Surgical, Sunnyvale, CA), have increased its popularity in surgical fields, especially surgical oncology, urology, and gynecology^1^. These benefits include, but are not limited to, smaller incisions, less pain, lower infection risk, and a shorter hospital stay^1,2^. Compared to conventional surgery, RAS presents more challenges for trainees, which include adjusting to a video view of anatomical structures rather than a direct view^3^, a lack of haptic feedback^4^, complex hand-eye coordination, the need for bimanual tool dexterity, and active foot coordination^5^. The establishment of a validated and standardized training protocol for RAS surgical trainees is crucial to ensure efficient and consistent training, patient safety, and high-quality outcomes.

The objective of this study is to develop linear models for evaluating performance and rate of learning RAS skills using features extracted from electroencephalogram (EEG) and eye-tracking data. These data were recorded from 26 subjects engaged in repeated RAS simulator tasks until successful completion (defined as a score of 70 out of 100). The analysis of the RAS skill acquisition did not adhere to a fixed timeframe, as the number of attempts varied among subjects.

### Available skill evaluation methods in RAS

Operative time (OT) is one of the measures for evaluating surgical learning progress^6^. While OT can indicate a surgeon’s proficiency and familiarity with an operation, utilizing this variable as a standalone criterion for performance evaluation may be misleading since this evaluation metric does not account for surgical outcomes^7^. Additional factors have been suggested to evaluate surgical performance, including intraoperative blood loss, length of hospital stay, functional outcomes^8,9^, and procedure-specific outcomes such as urinary incontinence and positive surgical margins following radical prostatectomy^10^. A more robust approach to assessing learning progress uses multidimensional analysis, which considers a variety of surgical performance markers^11^. Global Evaluative Assessment of Robotic Skills (GEARS) has been proposed as a tool to assess the RAS skills of trainees^12^. Robotic-Objective Structured Assessment of Technical Skills (R-OSATS) is an additional rating scale, evaluating key aspects such as respect for tissues, dexterity, fluency, knowledge, and accuracy^13^. Both GEARS and R-OSATS represent holistic assessment methods that provide a non-procedure-specific evaluation of trainees’ competencies, retrospectively covering all aspects of a task.

Lovegrove et al. have developed a modular training and assessment method, utilizing Healthcare Failure Mode and Effect Analysis^14^. In this approach, radical prostatectomy is segmented into seventeen distinct stages and sub-processes. Each sub-phase is then individually scored by experts. Competency in each stage is defined as acquiring a score of at least 4 out of 5 in all sub-processes consistently. However, modular assessment methods, while detailed, tend to be costly and less practical in live surgical settings. In addition, their results can be inconsistent and heavily dependent on raters’ subjective opinions, which may introduce bias. Despite the existence of some surgical performance tools like the Robotic Anastomosis Competency Evaluation for ureterovesical anastomosis (RACE)^15^, such methods are often task-specific and fail to encompass the entire surgical procedure^16^.

Computerized virtual reality simulations offer surgical trainees a safe environment to familiarize themselves with the robotic console and enhance their psychomotor skills without compromising the safety of patients^17,18^. These simulators have been shown to reduce the learning curve for surgical trainees^19^, leading to their widespread adoption in most training programs^20^. Yet, the development of objective and generalizable methods for evaluating performance and learning rates, essential for monitoring surgeons’ progress during training, continues to be a significant research gap. An ‘objective’ assessment technique not only evaluates performance but also aims to eliminate inconsistencies in evaluation. Currently, such a technique has not been fully developed within existing surgical practice protocols. In contrast, fields like aviation have significantly benefited from standardized, quality-assured training benchmarks. Pilots must demonstrate proficiency in numerous performance areas before being licensed to operate passenger planes^21^. However, this level of standardized, objective method has yet to be implemented in RAS surgical training.

### Proposed objective skill evaluation methods in RAS

Several studies have proposed objective surgical performance evaluation methods utilizing physiological data such as electroencephalogram (EEG)^22,23^, functional near-infrared spectroscopy (fNIRS)^24,25^, eye movement^26,27^, hands kinematics, and analysis of surgical videos^28–30^. While existing literature has utilized EEG for skill assessment, its focus has predominantly been on classifying experts and novices through EEG spectral analysis^31^, without considering the dynamic changes in EEG over time and across different brain areas. However, EEG has found application in performance evaluation in other fields, such as piloting and driving^32,33^. Eye-tracking, on the other hand, has been primarily used for workload evaluation^27^ and investigating the allocation of attentional resources^34,35^. Despite these uses, there remains a noticeable gap in the use of both EEG and eye-tracking for performance evaluation specifically in RAS training.

### The potential advantages of utilizing EEG and eye-tracking in RAS performance evaluation

The EEG’s high temporal resolution offers a dynamic perspective on cognitive processes during surgical tasks, going beyond what is possible with video processing of external movements. EEG directly measures neural mechanisms that are fundamental in skill learning and task execution, including attention levels, cognitive load, and decision-making processes. These aspects are vital for understanding surgical training and performance. Furthermore, EEG is capable of recording cortical activity, which is closely linked to learning processes. This cortical activity can change through practice and learning, reflecting neuroplasticity—the brain’s ability to reorganize itself by forming new neural connections in response to learning and experience^36^. EEG and eye-tracking can provide a multifaceted view of the surgical learning curve, capturing dimensions not visible in video data. EEG, for instance, can identify specific moments where a surgeon may experience a peak in cognitive load, which can be pivotal for modifying individual training programs.

### The potential limitations of utilizing EEG and eye-tracking in RAS performance evaluation

Collecting high-density EEG data, involving numerous channels (116 in this study), poses greater challenges than other methods like video analysis or hand movement tracking. The complexity arises from the technical demands of setting up many electrodes, potential signal losses due to electrode dropout, and the extensive pre-processing needed to ensure signal integrity. In contrast, video or motion tracking systems are generally more user-friendly, with fewer issues related to data loss. Furthermore, the practical application of EEG and other sensor-based methods is significantly limited by the difficulty in usage and potential disruptions caused by the equipment, a challenge not typically encountered with video-based methods. While video and motion tracking excel in providing spatial and temporal information about a surgeon’s techniques, high-density EEG offers unique insights into the cognitive processes behind surgical performance. Thus, despite its challenges, EEG remains an invaluable tool for a comprehensive performance assessment, encompassing both cognitive and physical aspects of surgery. Eye-tracking and EEG, with their distinct advantages, do not replace but rather complement video processing techniques. Together, they offer a more holistic understanding of the surgical learning curve.

### Potential use of machine learning approaches for surgical skill assessment

Information retrieved from hand movement kinematics, videos, EEG, and eye-tracking data has been used to develop deep convolutional neural networks, gradient boosting, and random forest models for surgical performance and skill evaluation^37–40^. The results from these approaches were promising. Developed machine learning algorithms, trained by physiological data, to identify predictors of performance have the potential to enable personalized learning and eventually automated performance feedback^41^.

This paper provides an exploratory analysis on the role of multi-source spatiotemporal signal processing in advancing automated surgical performance and learning rate evaluation.

## Results

Twenty-six subjects, having differing amounts of RAS practice, completed the Tubes (61 attempts), Suture Sponge (66 attempts), and Dots and Needles (66 attempts) tasks, achieving average performance scores of 71.47, 73.04, and 71.72, respectively. Linear random intercept models were developed for performance evaluation, while multivariate linear models were developed for learning rate evaluation. Age was not a significant predictor in these final models.

### Tubes task

Table 1 represents the results of the linear random intercept regression model analysis for evaluating the performance of the Tubes task. A one standard deviation increase in the average pupil diameter of the subject’s nondominant eye (standardized for each subject) was associated with an 8.13-point decrease in their performance score. This suggests that larger pupil sizes in the nondominant eye are linked to worse performance. In contrast, a one-standard deviation increase in the average temporal flexibility in Brodmann area 18 (BA 18) at beta band frequencies was associated with a 0.52-point performance improvement, suggesting that higher neural flexibility in this brain region enhances performance. In addition, a one standard deviation increase in rate of saccade was associated with a 5.87-point decrease in performance, indicating that more frequent saccades, compared to the individual’s average, are linked to lower performance scores.

**Table 1:** Results of a linear random intercept regression model for performance evaluation at the Tubes task with subject-wise standardized eye-tracking features.

Predictors	Estimate	Standard error	*p*-value
Nondominant eye’s average pupil diameter	−8.13	2.88	0.01
Average temporal flexibility in BA 18 at beta band frequencies	0.52	0.21	0.01
Rate of saccade	−5.87	2.74	0.04

Number of samples = 61; subject was a significant random effect (*p*-value = 0.02); pseudo R^2^ = 0.71; MAE: 6.79; RMSE: 8.79.

Table 2 illustrates the outcomes of the linear regression model analysis for the learning rate in the Tubes task. A one-standard deviation increase in the average temporal flexibility in BA 18 at theta-band frequencies was associated with a 0.59-point decrease in the learning rate, suggesting that larger temporal flexibility in BA 18 at theta-band frequencies is linked to poorer learning rates. Similarly, a one-standard deviation increases in the temporal flexibility in BA 46 at alpha-band frequencies corresponded to a 0.87-point decrease in learning rate, indicating that greater neural flexibility in this area of the brain is associated with a lower learning rate. Furthermore, each one-unit increase in initial performance score was associated with a 0.35-point decrease in learning rate, implying that subjects with higher initial scores tend to exhibit lower learning rates.

**Table 2:** Results of a multivariate linear regression model for learning rate evaluation at the Tubes task with subject-wise standardized eye-tracking features.

Predictors	Estimate	Standard error	*p*-value
Average temporal flexibility in BA 18 at theta-band frequencies at the first attempt	−0.59	0.26	0.03
Average temporal flexibility in BA 46 at alpha-band frequencies at the first attempt	−0.87	0.21	7 × 10^−4^
Performance at the first attempt	−0.35	0.086	5 × 10^−4^

Number of samples = 26; R^2^ = 0.64; MAE: 4.89; RMSE: 5.73.

### Suture Sponge task

Table 3 presents the results from the linear random intercept regression model for performance evaluation in the Suture Sponge task. A one-standard deviation increase in the average temporal flexibility in BA 10 at beta-band frequencies was associated with a 0.6-point improvement in the performance score for the suture sponge task, suggesting that enhanced neural flexibility in this area of the brain is associated with better performance. Likewise, a one-standard deviation increases in the average search information in BA 45 at theta-band frequencies corresponded to a 0.6-point increase in performance score.

**Table 3:** Results of a linear random intercept model for performance evaluation at the Suture Sponge task with subject-wise standardized eye-tracking features.

Predictors	Estimate	Standard error	*p*-value
Average temporal flexibility in BA 10 at beta-band frequencies	0.60	0.17	0.001
Average search information in BA 45 at theta-band frequencies	0.60	0.18	0.002

Number of samples = 66; subject was a significant random effect (*p*-value = 0.004); pseudo R^2^ = 0.75; MAE: 5.43; RMSE: 6.83.

Table 4 displays the findings from the linear regression model analysis for the learning rate in the Suture Sponge task. A one-standard deviation increase in the average search information in BA 45 at theta-band frequencies was associated with a 1.08-point decrease in the learning rate, suggesting that higher search information in this area and frequency band correlates with a lower learning rate. Similarly, a one-standard deviation increases in the average temporal flexibility in BA 45 at theta-band frequencies corresponded to a 0.31-point decrease in learning rate, indicating that increased neural flexibility in this area is associated with a reduced learning rate. Conversely, a one-standard deviation increase in the average search information in BA 19 at gamma-band frequencies was associated with a 1.19-point increase in the learning rate.

**Table 4:** Results of a linear regression model for learning rate evaluation at the Suture Sponge task with subject-wise standardized eye-tracking features.

Predictors	Estimate	Standard error	*p*-value
Average search information in BA 45 at theta-band frequencies at the first attempt	−1.08	0.25	3 × 10^−4^
Average temporal flexibility in BA 45 at theta-band frequencies at the first attempt	−0.31	0.08	8 × 10^−4^
Average search information in BA 19 at gamma-band frequencies at the first attempt	1.19	0.44	0.01

Number of samples = 26; R^2^ = 0.71; MAE: 5.71; RMSE: 7.27.

### Dots and Needles task

Table 5 presents the outcomes from the linear random intercept regression model analysis for performance in the Dots and Needles task. A one-standard deviation increase in the average search information in BA 37 at gamma-band frequencies was associated with a 1.35-point decrease in the performance score for this task. In addition, a one-standard deviation increase in the entropy of the nondominant eye’s pupil diameter was associated with a 4.68-point decrease in performance score.

**Table 5:** Results of a linear random intercept regression model for performance evaluation at the Dots and Needles task with subject-wise standardized eye-tracking features.

Predictors	Estimate	Standard error	*p*-value
Average search information in BA 37 at gamma-band frequencies	−1.35	0.39	0.0001
Shannon entropy of nondominant eye’s pupil diameter	−4.68	1.82	0.01

Number of samples = 66; subject was a significant random effect (*p*-value = 0.005); pseudo R^2^ = 0.75; MAE: 6.44; RMSE: 8.03.

Table 6 showcases the results from the linear regression model analysis for the learning rate in the Dots and Needles task. A one-standard deviation increase in the average search information in BA 45 at beta-band frequencies was associated with a 1.92-point increase in the learning rate value for this task. Similarly, a one-standard deviation increases in the average search information in BA 40 at alpha-band frequencies corresponded to a 1.45-point increase in learning rate. In addition, a one-standard deviation increase in the average temporal flexibility in BA 41 at theta-band frequencies was associated with a 0.41-point increase in learning rate.

**Table 6:** Results of a linear regression model for learning rate evaluation at Dots and Needles with subject-wise standardized eye-tracking features.

Predictors	Estimate	Standard error	*p*-value
Average search information in BA 45 at beta-band frequencies at the first attempt	1.92	0.48	0.0006
Average search information in BA 40 at alpha-band frequencies at the first attempt	1.45	0.37	0.0009
Average temporal flexibility in BA 41 at theta-band frequencies at the first attempt	0.41	0.16	0.021

Number of samples = 26; R^2^ = 0.69; MAE: 5.69; RMSE: 7.15.

**Fig. 1:**
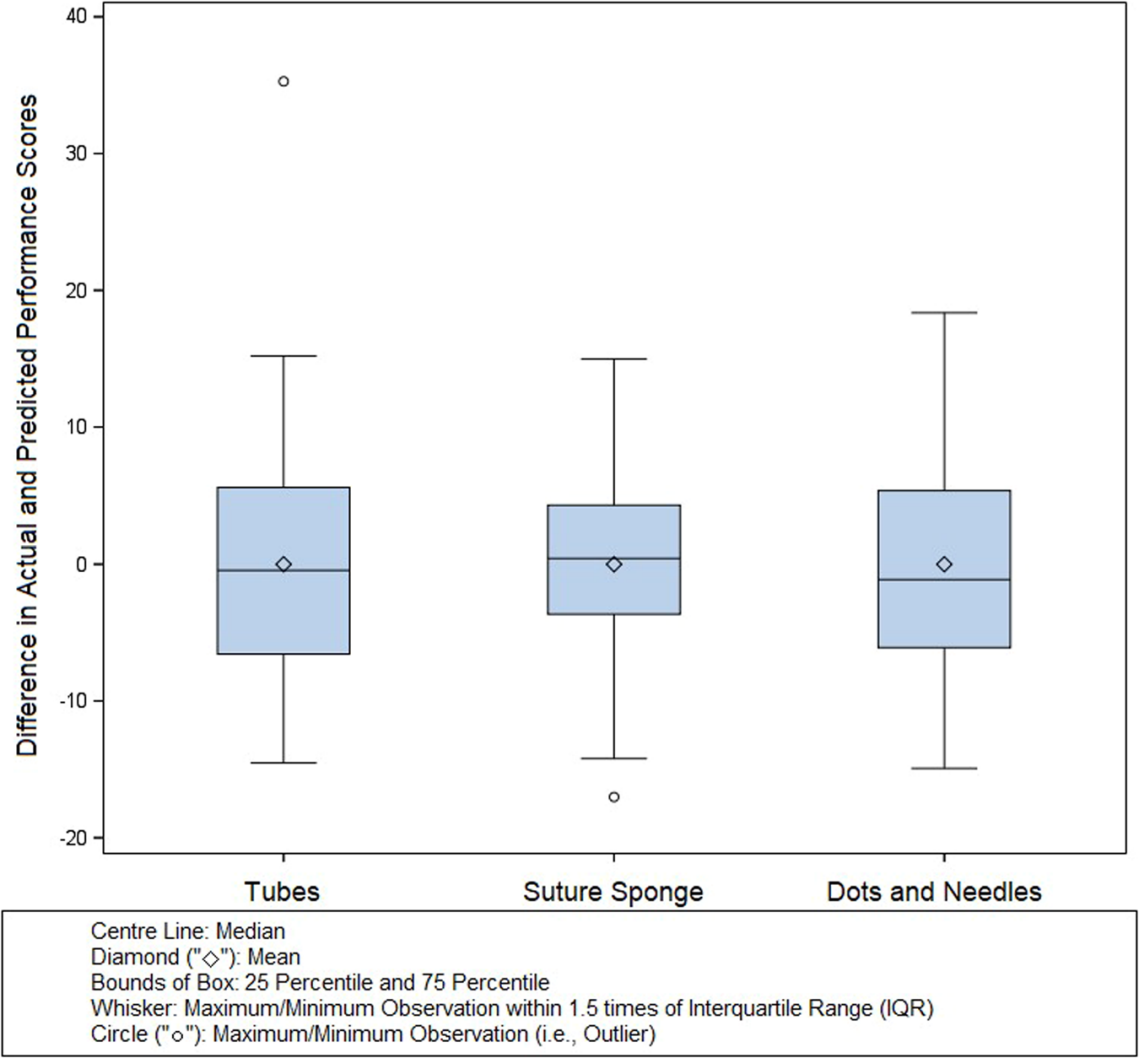
Representation of differences between predicted and actual performance scores in Tube, Suture Sponge, and Dots and Needles tasks. This box plot utilizes whiskers to represent the maximum and minimum observations within 1.5 times the interquartile range (IQR), rather than mean ± standard deviation. This method was chosen as it more accurately reflects the distribution and variability of our prediction, particularly in the presence of outliers.

We created boxplots to illustrate the differences between predicted and actual performance scores (Fig. 1). The analysis reveals that both the mean and median differences are close to zero. Moreover, for most samples, the absolute difference between actual and predicted performance scores was less than 10. These findings indicate that our performance evaluation models for the three tasks are reasonably accurate.

#### Effect of subject-wise standardization of eye-tracking features

Supplementary Information details the outcomes of the linear random intercept regression models for performance evaluation and the linear regression analysis for learning rate evaluation, conducted without subject-wise standardization of features (Supplementary Information). The results indicate that subject-wise standardization of eye-tracking features marginally enhanced the R^2^ values for both performance (0.17 increase for the Tubes task, 0.04 increase for the Dots and Needles task) and learning rate evaluations (0.09 increase for the Dots and Needles task).

#### Relationship between hours of experience with RAS and performance

Pearson correlation analysis was conducted to examine the relationship between subjects’ hours of RAS practice and their performance. The results revealed no significant correlation between RAS practice hours and performance in the Tubes task (Pearson correlation; *p*-value = 0.20), Suture Sponge task (Pearson correlation; *p*-value = 0.07), and Dots and Needles task (Pearson correlation; *p*-value = 0.85).

#### Relationship between performance and mental workload

The Pearson correlation between performance and mental workload was not significant for the Tubes task (Pearson correlation; *p*-value = 0.37), Suture Sponge task (Pearson correlation; *p*-value = 0.79), and Dots and Needles task (Pearson correlation; *p*-value = 0.97).

## Discussion

### Tubes

Our findings indicate a negative association between the average pupil diameter of the nondominant eye and performance in the Tubes task, as shown in Table 1. This result aligns with the literature^42,43^, supporting the notion that pupillometry, the measurement of pupil diameter, is a reliable marker of mental workload and performance^42–44^. Pupil dilation has been shown to be associated with higher workloads and lower performance scores^42^.

To successfully complete the Tubes task, subjects must consciously track targets, drive needles through them, visually anticipate upcoming targets, enhance hand-eye coordination, and drive the needle through the yellow side of the target. The significant correlation between performance and rate of saccade identified in this study (Table 1) is consistent with these required skills. Saccades are known to be essential for attention^45,46^, and both consciousness (perceptual awareness required for engaging with the Tubes task) and attention are critical for making timely and accurate decisions in this task.

Average temporal network flexibility in Brodmann area 18 (BA 18) at beta-band frequencies showed a positive association with performance in the Tubes task (Table 1). Functional MRI studies have indicated that BA 18 plays a role in basic visual functions, such as attention and pattern detection, and in processing visuo-spatial information^47,48^. In addition, brain oscillations in the beta-band frequencies are associated with logical and conscious thinking^49^. The selection of this feature as a performance predictor in our study may show the importance of attention and visuo-spatial information processing in the Tubes task. Therefore, greater flexibility in BA 18 at beta-band frequencies may enhance attention and adaptation to new visual stimuli, leading to quicker decision-making and ultimately improved performance in the Tubes task.

Performance at the first attempt was identified as a predictor of learning rate in the Tubes task, possibly due to the high standard deviation (SD) of performance scores in this task (SD = 16.3).

### Suture Sponge task

To successfully complete the Suture Sponge task, subjects need to skillfully control needles and navigate them through a deformable object. Since the object is deformable and its interior is invisible, subjects often need to correct their hand motions for accurate needle insertion and extraction, while also choosing appropriate movements based on the needle and target positions. The association between selected EEG features and performance in this task (Table 3) aligns with these requirements. Functional MRI studies have shown that Brodmann area 10 (BA 10) is involved in various memory functions, executive control, error processing, and decision-making^50–54^, while BA 45 is associated with reasoning processes and working memory^51,55^. As a result, increased flexibility in BA10 at beta-band frequencies and enhanced search information in BA 45 at theta-band frequencies may be associated more efficient memory retrieval, error processing, and decision-making, thereby leading to better performance in the Suture Sponge task.

Our findings showed that BA 45 functioning plays a key role not only in performance evaluation but also in the learning rate evaluation of the suture sponge task (Tables 3 and 4). Its search information and flexibility at theta-band frequencies were associated with the learning rate (Table 4), aligning with literature that underscores BA 45’s involvement in reasoning processes and working memory^51,55^. In addition, gamma-band frequencies are associated with perception, cognitive processes, attention, working memory, and information integration^56,57^. BA 19, known for its role in spatial working memory, visual memory recognition, and visuo-spatial information processing^48,58,59^, also showed a connection with learning rate through its search information in gamma-band frequencies (Table 4), representing the skills necessary for the successful completion of the Suture Sponge task.

### Dots and Needles task

Our results showed that entropy of the nondominant eye’s pupil diameter is negatively associated with performance (Table 5). Since entropy of eye’s pupil diameter has been proposed in prior studies as a measure of visual scanning efficiency^60^, this association may indicate that fewer resources are available to perform the task when the entropy is higher. Hence, this finding may be interpreted as suggesting that lower cost of retrieving information from the visual system may be associated with a better performance^61^.

The EEG features selected for performance evaluation in the ‘Dots and Needles’ task (Table 5) align well with the task’s requirements. This task requires subjects to (1) develop hand-eye coordination skills for precise needle placement and manipulation through soft objects, and (2) precisely detect target positions and execute needle insertion and extraction. Functional MRI studies have shown that BA 37 plays a crucial role in complex visual motion processing^62^, structural judgment of familiar objects^63^, and visual memory processes^59^. The observed association between EEG features and performance in ‘Dots and Needles’ suggests that higher search information levels may reflect an increased need for visual and cognitive information processing in BA 37, which could potentially reduce performance.

The observed associations in Table 6—between learning rate and search information in BA 45 and BA 40, as well as between learning rate and temporal flexibility in BA 41—align with the required skills for the ‘Dots and Needles’ task. Functional MRI studies indicate that BA 40 plays a role in various activities, including visually guided grasping, visuomotor transformation/motor planning, response to visual motion, and working memory^64–68^, and BA 41 is linked to working memory^69^.

### Effect of subject-wise standardization of eye-tracking features

Comparing the performance and learning rate evaluation models with subject-wise standardization (Tables 1 to 6) against those without such standardization (Supplementary Information), reveals that subject-wise standardization reduces the impact of individual variances among subjects. As a result, the standardized features more accurately reflect skill differences as opposed to variations in subjects’ individual characteristics.

### Relationship between practice hours and performance

This study found no significant correlation between subjects’ hours of RAS experience and task performance, which could be attributed to the quality of practice rather than its quantity. Effective performance improvement likely depends on proper execution of RAS tasks. Moreover, it has been shown that extended breaks between practice sessions might disrupt functional brain networks, affecting performance^70^. It’s also worth noting that inefficient practice, despite increasing the total practice hours, may not necessarily lead to performance enhancement.

### Relationship between performance and mental workload

Our study revealed no significant correlation between performance and mental workload. Mental workload represents the balance between a person’s cognitive capacity and the demands a task imposes on them^71,72^. Acquiring new skills typically involves enhancing both performance and mental workload management^22,73,74^. Previous research indicates that during skill acquisition, mental workload may continue to decrease even after achieving a passing performance score^75^. Therefore, the absence of a significant correlation in our study might imply that some subjects were still refining their RAS skills beyond achieving passing scores, indicative of ongoing improvements in their mental workload management.

### Practical implications of the findings

The findings establish a basis for an objective evaluation of the performance and learning rate of RAS trainees. The developed models, once validated for a broader population and surgical tasks, could be used in surgical residency programs to improve the RAS skill acquisition process in three possible ways: (1) They provide objective, unbiased assessments of RAS trainees’ performance without needing an expert RAS surgeon’s presence during practice sessions. This approach reduces training costs and offers immediate performance feedback, allowing trainees to correct mistakes more efficiently and shorten the learning process. Consequently, training programs can admit more RAS trainees and expedite their graduation, streamlining the overall training procedure. In addition, this model enables training of more RAS surgeons annually, increasing the number of patients who can benefit from RAS technology. Hospitals will also benefit, as RAS typically involves shorter hospital stays and fewer surgical complications compared to traditional surgery methods^76,77^; (2) The learning rate evaluation models, based on data from the first attempt, enable RAS training programs to predict specific trainees’ learning rates. This information allows programs to either select better RAS learners or plan effective strategies to enhance learning for those who progress more slowly; (3) Such performance and learning rate evaluation methodologies could be used for a broader range of surgical tasks, particularly those that are similar to actual surgical operations.

### Limitations of the study

Several limitations may impact the generalizability of the findings of this study. First, the moderate R^2^ values of the learning rate evaluation models (0.64, 0.71, and 0.69 for the Tubes, Suture Sponge, and Dots and Needles, respectively) might be attributed to limited sample sizes. Second, as the study was conducted within a single U.S. health system, its findings may not be applicable to other institutions, specialties, or countries. Further validation of the models is needed, incorporating data from a more diverse group of subjects across various hospitals and specialties, and involving different surgical tasks. Third, exploring potential nonlinear relationships between learning rate and the features requires more attempts per subject and analysis using nonlinear regression models. Lastly, the inherent challenges associated with the use of EEG and other sensor-based techniques, coupled with the potential disruptions caused by the equipment, limit their practical application.

## Methods

This study was conducted in accordance with relevant guidelines and regulations and was approved by the Roswell Park Comprehensive Cancer Center (RPCCC)’s Institutional Review Board (IRB; I-241913). The IRB issued a waiver of documentation of written consent, and the subjects were given a research study information sheet and provided verbal consent.

### Subjects

The experiments involved a group of 26 subjects, and the demographics and relevant experiences of all subjects are detailed in Table 7. The ‘Hours of RAS Experience’ column reflects each subject’s experience hours. The subjects themselves provided this information. Each subject was required to perform every task at least twice, aiming for a minimum score of 70 out of 100 to qualify as a successful attempt. If the required score was not achieved within the first two attempts, they continued to try until meeting the benchmark.

**Table 7:** Demographics of subjects and number of task attempts.

Subject	Age	Gender/Sex^a^	Dominant hand^b^	Dominant eye^b^	Specialty, position	Hours of RAS practice	Number of attempts		
							Tubes	Suture Sponge	Dots and Needles
1	32	M	R	R	Urology, fellow	500	2	2	2
2	36	M	R	L	Urology, fellow	100	2	2	2
3	22	F	R	R	No specialty, pre-medical student	0	2	2	2
4	26	M	R	R	No specialty, pre-medical student	0	3	2	2
5	33	M	R	R	Gynecology, fellow	120	2	2	5
6	35	M	R	R	Urology, fellow	100	2	2	2
7	33	M	R	R	Gynecology, resident	10	2	2	2
8	61	M	R	R	Thoracic, surgeon	30	2	2	2
9	44	M	R	L	Gynecology, RAS surgeon	500	3	2	2
10	24	F	R	L	No specialty, pre-medical student	0	3	4	4
11	34	F	R	L	Gynecology, fellow	80	2	2	2
12	44	M	R	R	Urology, RAS surgeon	More than 1000	2	2	2
13	67	M	R	R	Urology, RAS surgeon	More than 1000	3	2	2
14	23	F	R	L	No specialty, pre-medical student	0	2	3	2
15	47	M	R	R	Urology, RAS surgeon	More than 1000	2	2	2
16	22	F	R	R	No specialty, pre-medical student	0	2	2	2
17	36	F	R	R	No specialty, Scientist	0	4	9	2
18	23	F	R	R	No specialty, pre-medical student	0	2	2	2
19	28	M	R	R	No specialty, pre-medical student	0	2	3	5
20	32	M	R	R	Gynecology, resident	15	2	3	3
21	20	M	R	L	No specialty, pre-medical student	0	4	2	2
22	34	M	R	R	No specialty, researcher	0	2	2	2
23	32	F	R	R	Gynecology, fellow	40	2	2	2
24	42	M	L	L	Head and neck, surgeon	55	2	2	2
25	54	M	R	R	Oncology, oncologist	0	2	3	7
26	39	M	R	L	Urology, fellow	0	3	3	2

^a^In this study, all participants’ gender identities aligned with their biological sex. Male (M), Female (F).

^b^Right (R), left (L).

### Skill level of subjects

This manuscript does not aim to classify skill levels based solely on hours of experience, recognizing that proficiency can vary significantly across different tasks. Such categorization would require specific assessments beyond the scope of this study. For general categorization purposes, RAS surgeons in Table 7 are considered RAS experts and typically act as primary surgeons. Surgical fellows are typically estimated to be competent, whereas residents are often viewed as beginners. In our categorization, oncologists, researchers, students, and scientists are generally labeled as novices. It’s important to note that thoracic surgeons and head and neck surgeons, despite their expertise in other surgical areas, are classified as novices or beginners in RAS for this study. These categories are broad and should not be taken as a substitute for detailed skill assessment.

### Recruitment method

Subjects were invited to the study via email or verbal invitation. Subjects included surgeons, fellows, residents, pre-medical students, and/or scientists at Roswell Park Cancer Institute.

### Data recording set up

**Fig. 2:**
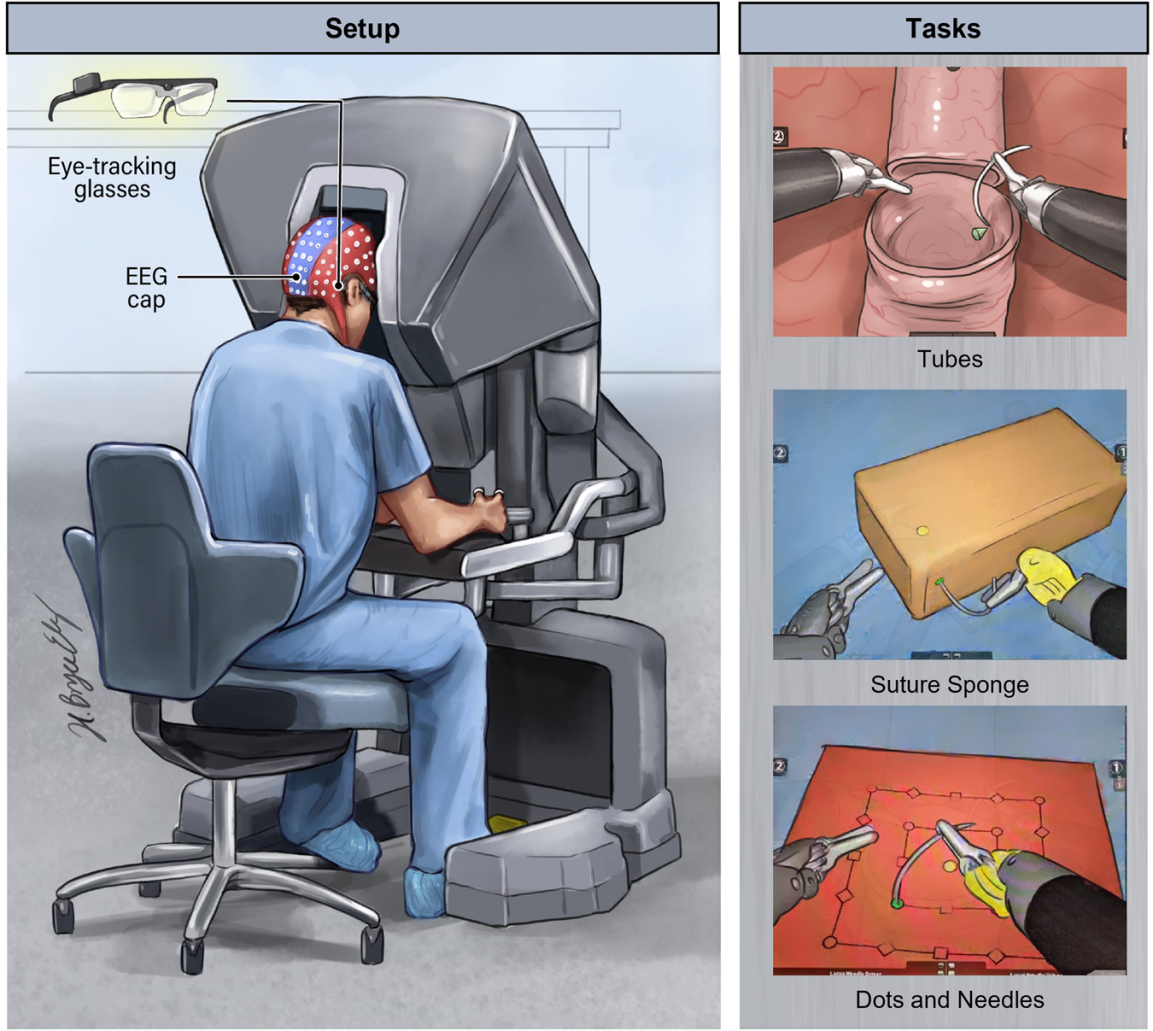
Representation of a subject completing three tasks on the da Vinci simulator while wearing an EEG headset and eye-tracking glasses. This figure was developed by the ATLAS illustrator Shared Resource at RPCCC, using the Adobe Illustrator software.

The da Vinci® Skills Simulator™ (developed in collaboration with Mimic® Technologies, Inc., Seattle, WA, USA) has two instruments attached to mechanical arms and a camera arm. The subject operates the arms while sitting at a computer console (Fig. 2). The Tubes, Suture Sponge, and Dots and Needles tasks were completed by subjects using the da Vinci® Skills Simulator™. A 124-channel EEG headset from AntNeuro® was used to record EEG data at a frequency of 500 Hz, using Cz as the reference channel. Simultaneously, Tobii® eyeglasses were utilized to record eye-tracking data at a frequency of 50 Hz, as illustrated in Fig. 2. Due to the poor quality of signals recorded from the F8, POz, AF4, AF8, F6, FC3, M1, and M2 channels, data from these channels were excluded from the study. The analysis was conducted on the signals from the remaining 116 channels.

### Tasks and the purpose of each task

Subjects were instructed to watch a training video before performing the task. The Tubes, Suture Sponge, and Dots and Needles tasks with their highest level of complexity were included (Fig. 2). Subjects always conducted the tasks in the same order.

#### Tubes task

Subjects practiced tissue manipulation and needle driving skills that will be encountered as part of a urethral anastomosis (i.e., a challenging portion of a radical prostatectomy operation). Both simulator instruments were used to manipulate two vessels to facilitate needle driving. Subjects were instructed to insert the needle through the yellow side of the target and then guide it out from the black side. The task was to continue driving the needle tip through the yellow target until it changed to green.

#### Suture Sponge task

Subjects were trained to enhance their dexterity and precision in manipulating a needle through a deformable object. This involved controlling the needle during its transfer between instruments, as well as during insertion and extraction through various pairs of targets. These targets were placed on the edge of a sponge, with random variations in their positions and sizes.

#### Dots and Needles task

Subjects were taught to perform challenging needle throws through a soft, flexible object. The task required them to insert and accurately guide a needle through several pairs of targets, each varying in spatial distance and position. Upon the first target changing to green, the subjects had to skillfully rotate their wrist to drive the needle tip through the second yellow target, continuing until it too turned green.

### Attempts

Each subject performed every task a minimum of two times. If they did not attain a passing score of 70 out of 100 on at least one of these two attempts, they continued repeating the task until the passing score was achieved.

### Mental workload

At the end of each attempt, subjects completed the Surgery Task Load Index (SURG-TLX) questionnaire to assess their mental workload. The SURG-TLX is a tool comprising six domains that measure perceived workload^78^. These domains are *mental demands*: the level of mental effort required during task completion; *physical demands*: the level of physical effort required during task completion; *temporal demands*: the level of time pressure felt in completing the task; *task complexity*: the degree of difficulty of the task; *situational stress*: the level of stress or anxiety experienced while completing the task; *and distractions*: the degree of distraction from the surrounding environment. Each domain is scored on a scale from 1 to 20, where 1 indicates the lowest and 20 indicates the highest level. The overall mental workload score was calculated by summing the scores from all six domains.

### Performance scores

After the subject completed each attempt of the tasks, the simulator generated a single score between 0 and 100 based on their performance, where 0 indicated no acceptable performance and 100 represented performance that satisfied all necessary standards. To determine the performance score, the simulator program uses the following metrics: the time required to complete the exercise (measured in seconds); economy of motion: the total distance traveled by all instruments (measured in centimeters); instrument collisions: the total number of instrument-on-instrument collisions; excessive instrument force: the total time an excessive force was applied to the instrument (measured in seconds); instruments out of view: the total distance traveled by instruments outside of the user’s field of view (measured in centimeters); master workspace range: the radius of the user’s working volume on master grips (measured in centimeters); drops; and missed targets.

### Learning rate

The learning rate was defined as the change in performance score per additional attempt. The learning rate was calculated for subjects performing each task as the slope of a linear regression fitted on the performance scores across attempts.

### EEG Pre-processing

Signals from 116 EEG channels underwent artifact decontamination through blind source separation and topographical principal component analysis within the Advanced Source Analysis (ASA) framework. The framework has been developed by ANT Neuro Inspiring Technology Inc., Netherlands. In this study, the EEG artifact decontamination was carried out in five distinct steps: (1) The EEG data were re-referenced to the ‘common average reference,’ which involves averaging the signals from all channels used in the study^79^. (2) A 60 Hz notch filter was applied to eliminate line noise. (3) The data were then processed with a band-pass filter, ranging from 0.2 to 250 Hz, with a steepness of 24 dB/octave. (4) Facial and muscle activity-related artifacts were detected and removed using ASA, followed by a visual inspection of individual EEG data segments for any remaining artifacts^79^. (5) Finally, the Spatial Laplacian technique, known for emphasizing sources at small spatial scales, was utilized to reduce the effects of volume conduction on coherence calculations^80^.

After decontaminating the EEG data, they were utilized to extract search information and temporal network flexibility features in theta (4–8 Hz), alpha (8–12 Hz), beta (13–35 Hz), and gamma (35–65 Hz) frequency bands, spanning 21 Brodmann Areas (BA).

### Distribution of EEG channels across Brodmann Areas

Each EEG channel was assigned to a specific BA based on its approximate position over the area. The correspondence between EEG channels and BAs was determined using Brodmann’s Interactive Atlas (http://www.fmriconsulting.com/brodmann/Interact.html) and the Brain Master software (http://www.brainm.com/software/pubs/dg/BA_10-20_ROI_Talairach/). This assignment process categorized the 116 EEG channels into the 21 BAs, as detailed in Table 8.

**Table 8:** List of EEG channels roughly above each Brodmann Area.

BA	Channels	BA	Channels	BA	Channels
1	C4, CCP4h	2	C3, CP3, CCP3h, CPP3h	5	Cz, CP1, CP2, C1, C2, CCP1h, CCP2h, CPP1h, CPP2h
6	FC1, FC2, FCz, FC4, FCC3h, FCC4h, FCC2h, FCC1h	7	Pz, P1, P2	8	F4, F3, Fz, F1, F2, AFF1, AFF2, FFC3, FFC4, FFC1, FFC2
9	AF3, AFz	10	AFp3h, AFp4h, FPz, FP3, FP4, FP1, FP2	18	O1, O2, I1, I2, OI1h, OI2h, POO9h, POO10h
19	PPO2, PPO1, POO3h, POO4h, PO3, PO4, PO7, PO8	20	FT9, FT10, PO9, P9, FTT9h, FTT10h, PPO9h	21	TP7, TP8, TPP10h, TTP8h, TPP7h, TPP8h, T8, TPP9h
37	PO10, P10, PPO10h, P7, P8	39	P3, P4, P5, P6, PPO5h, PPO6h	40	CP5, CP6, CP4, CPP5h, CPP6h
41	C6, CCP6h	42	CCP5h, TTP7h, T7, C5	44	FC6, FC5, FCC6h
45	FFT8h, FFT7h	46	AFF5h, AFF6h, AF7, F5, FFC5h, FFC6h, FCC5h	47	FTT7h, FTT8h, F7, FT7, FT8

### Traditional names for numbered Brodmann’s areas (BAs)

BAs 1 and 2 represent the primary somatosensory cortex; BA 5 is known as the pre-parietal (somatosensory association) cortex; BA 6 encompasses the premotor and supplementary motor cortices; BA 7 is identified as the superior parietal (somatosensory association) cortex; BA 8 is intermediate frontal; BAs 9 and 10 correspond to the dorsolateral prefrontal cortex; BA 18 is the secondary visual cortex; BA 19 is the associative visual cortex; BA 20 is the inferior temporal cortex; BA 21 is middle temporal cortex; BA 37 is known as occipitotemporal; BA 39 is angular (i.e., an area in the parietal lobe); BA 40 is supramarginal (i.e., a portion of the parietal lobe); BAs 41 and 42 are the anterior and posterior transverse temporal areas, respectively; BA 44, also known as opercular (i.e., refers to the frontal, temporal, or parietal operculum, which together cover the insula); BA 45, the triangular area, is a part of Broca’s area on the left hemisphere; BA 46 is the middle frontal area; and BA 47 is referred to as orbital (i.e., an area of the prefrontal cortex).

### Extraction of search information feature using EEG data

**Fig. 3:**
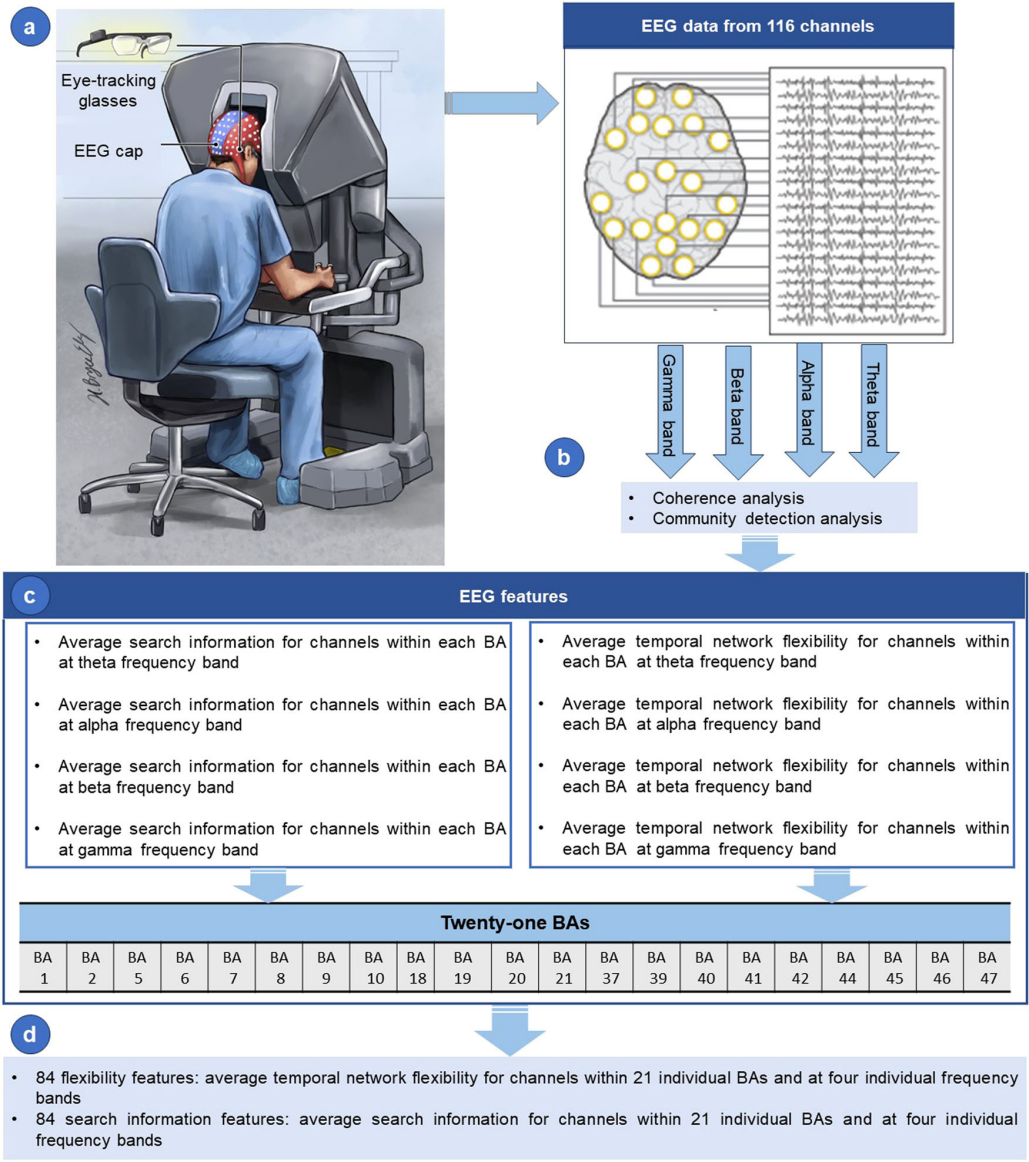
Feature extraction from EEG data across brain areas and frequency bands. (**a**) Data recording set up. (**b**) EEG data analysis. (**c**) Extraction of EEG features from 21 individual Brodmann Areas (BAs) across four frequency bands. (**d**) The feature extraction process results in a comprehensive set of 168 distinct EEG features. Part (**a**) of this figure was developed by the ATLAS illustrator Shared Resource at RPCCC, using the Adobe Illustrator software.

Search information is the amount of information (measured in bits) required to pass the shortest, and presumably the most efficient path between two nodes of a network^81–83^. The search information feature was extracted using the adjacency matrix, commonly known as the functional brain network, of each EEG recording^81,82^ and the Brain Connectivity Toolbox (https://sites.google.com/site/bctnet/). The adjacency matrix is a network that mathematically illustrates the functional connections between the various brain areas involved in information processing^84^. The entries in the adjacency matrix represent the average magnitude coherence (MC) across specific frequency bands. Magnitude coherence is a measure of the statistical similarity between two time series, in this case, the EEG signals from different channels. The MC values are calculated for each pair of EEG channels *i* and *j* (Γ = (*Γ*_*ij*_) ∈ℜ^*N*X*N*^, with *i* and *j* ranging from 1 to *N*, where *N* is the number of EEG channels) and are assessed over designated frequency bands. These values were obtained using coherence analysis in this study^85^. Finally, 84 search information features were generated by averaging the extracted feature for channels within each of the 21 BAs, across four band frequencies (Fig. 3).

### Extraction of temporal network flexibility feature using EEG data

The temporal network flexibility (*f*) of each network node is proportional to the number of times the node changed its network community assignment over time^86^. A network community is described as a subset of network nodes with denser connections between themselves compared to connections with other nodes in the network^87^. Temporal network flexibility has been proposed as a functional brain network feature that changes with learning^88^, surprise, and fatigue^86^. This feature has also been proposed for evaluating the mental workload of surgeons conducting surgical tasks^89^.

To calculate the temporal network flexibility feature, an adjacency matrix (i.e., functional brain network) was extracted for every one-second window of EEG data recording. Then, the modularity metric associated with each adjacency matrix was extracted using the “community Louvain” function of the Brain Connectivity Toolbox. This metric measures how well nodes are assigned to communities. To detect network communities, modularity was maximized using a Louvain-like locally “greedy” algorithm^90,91^. This process was repeated 100 times using a consensus iterative algorithm to identify a single consistent representative partition from all partition sets based on statistical testing in comparison to the ‘Newman-Girvan (NG)’ null network^91,92^. The output of modularity maximization is the community assignment of EEG channels for each 1-second window EEG. The community assignment of each EEG channel is the community that the EEG channel was assigned to (e.g., if three communities were detected for an adjacency matrix, the community assignment of each node is an integer from one to three). The community assignments of EEG channels across 1-second windows were used as elements of the partition matrix *A* ∈ ℜ^*N*X*T*^. The elements of the partition matrix ${A}_{i,t}\,\in\, \left\{1...g\right\}$ displayed the communities (*g*) to which brain area *i* (EEG channels; 1 to *N*, where *N* = 116) was assigned at time *t* (second; *t* = 1 to *T*, where *T* denotes recording duration).

Finally, the partition matrix was used in the flexibility function of the Network Community Toolbox (http://commdetect.weebly.com/)^93^ to calculate the temporal network flexibility of each channel as Eq. 1.

1 $${f}_{i}=1-\frac{1}{T-1}\mathop{\sum }\limits_{t=1}^{T-1}\delta \left({A}_{i,t},{A}_{i,t+1}\right)$$

where, ${f}_{i}$ is the temporal network flexibility of channel *i*, defined as the number of times that brain area *i* changed its community assignment across successive 1-s time windows. High values of ${f}_{i}$ indicate frequent changes in community assignments (high temporal flexibility), while low values suggest stable assignments (low temporal flexibility)^86,93^. In Eq. 1, ‘*A*’ is the partition matrix, and ‘*T*’ is the recording duration. The $\delta ({A}_{i,t},{A}_{i,t+1})$ represents a binary function used to determine whether the community assignment of brain area *i* changes between two successive time windows *t* and *t* + 1. The function *δ* takes the value 1 if there is a change in the community assignment of brain area *i* from one time window to the next. If there is no change in community assignment, *δ* takes the value 0. Finally, the average of the extracted temporal network flexibility for channels within each BA was calculated at four band frequencies, resulting in a total of 84 temporal network flexibility features, corresponding to 21 BAs and four frequency bands (Fig. 2).

### Extraction of eye-tracking features

**Fig. 4:**
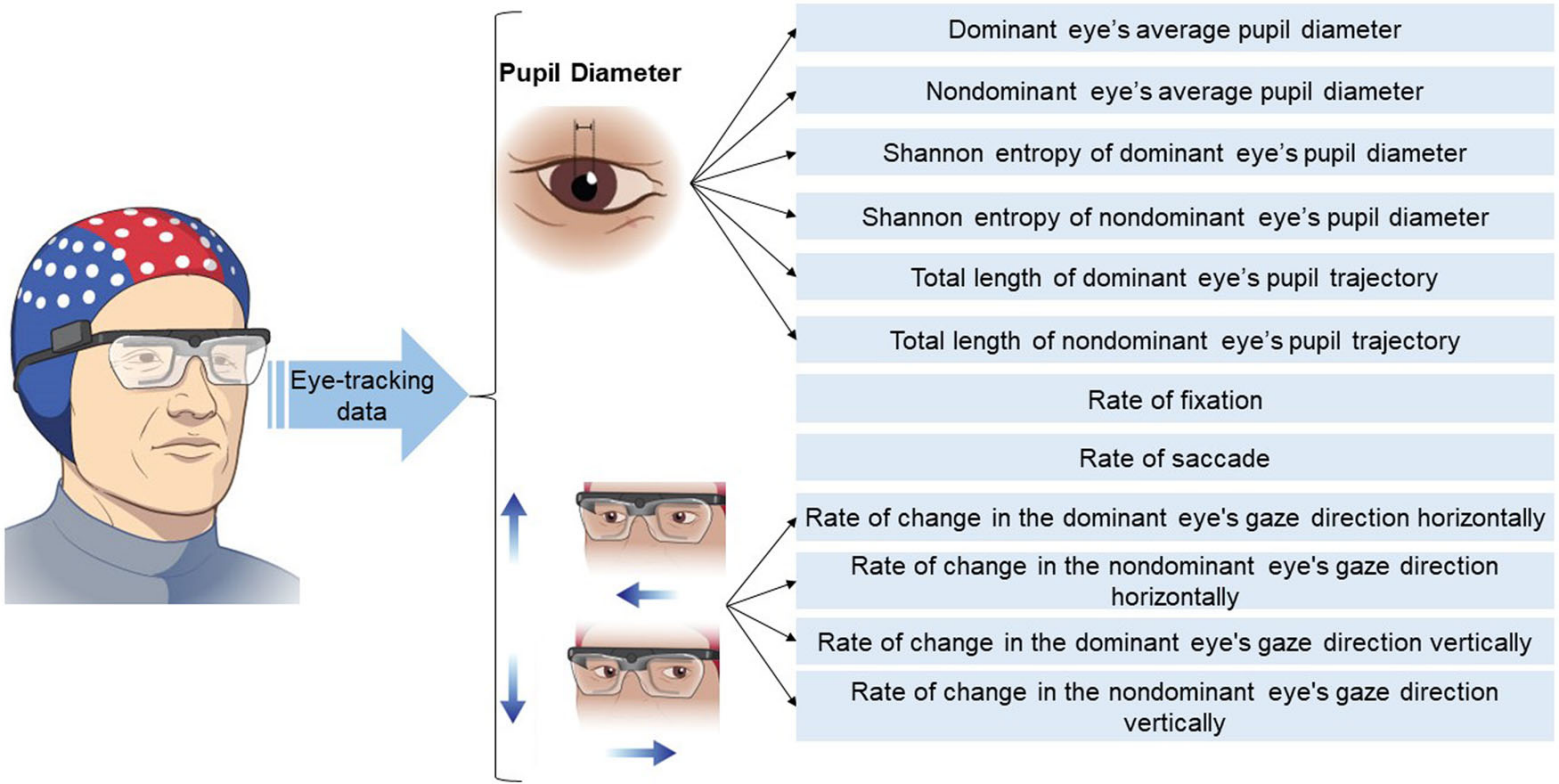
Feature extraction from eye-tracking data. Eyeglasses parts of this figure were developed by the ATLAS illustrator Shared Resource at RPCCC, using the Adobe Illustrator software.

Tobii Pro Lab © was used to process eye-tracking data. A moving average filter with a window size of three points was applied to reduce noise in eye-tracking data. A velocity-threshold identification fixation filter with a threshold of 30 degrees per second was used to identify fixation and saccadic points. Features extracted from eye-tracking data were defined in Table 9 and Fig. 4. Extracted eye-tracking features were then standardized for each subject (i.e., subject-wise standardization). Subject-wise standardization: for each subject, the mean (µ) and standard deviation (σ) of each eye-tracking feature (X), within the task, are calculated, and then the mean value is extracted from each eye-tracking feature, and the result is divided by the standard deviation value ((X − µ)/σ)^94^.

**Table 9:** Definition of eye-tracking features.

Eye-tracking feature	Definition	Number of extracted features
(1) Rate of fixation	Number of eye-tracking time points that fell below the threshold of 30 degrees per second divided by the number of total time points of the recording	1
(2) Rate of saccade	Number of eye-tracking time points with an angular velocity higher than the threshold of 30 degrees per second, divided by the number of total time points of the recording	1
(3) Average pupil diameter	Average pupil diameter of each eye throughout a recording	2
(4) Shannon entropy of pupil diameter	Shannon entropy: the average rate at which information is produced by a stochastic source of data. For a signal *X*(*t*), the Shannon entropy *S*(*X*) is calculated as $S\left(X\right)=-\mathop{\sum }\limits_{i=1}^{N}p\left({x}_{i}\right){\log }_{2}\left(p\left({x}_{i}\right)\right)$ where *p*(*x*_*i*_) is the probability of obtaining the value *x*_*i*_. This feature was calculated for both eyes.	2
(5) Rate of changes in the eye’s gaze direction	Calculation: The total number of time points at which the direction of the eye changes is divided by the total number of time points. This feature was calculated for both eyes and both directions (horizontal and vertical).	4
(6) Total length of eye pupil trajectory	For pupil trajectory in three dimensions [$X(i),{Y}(i),Z(i)$] with *N* points, the total length can be calculated as: $L=\mathop{\sum }\limits_{i=1}^{N-1}\sqrt{{(X(i+1)-X(i))}^{2}+{(Y(i+1)-Y(i))}^{2}+{(Z(i+1)-Z(i))}^{2}}$ This formula calculates the Euclidean distance (straight-line distance) between each pair of successive points and sums these distances to get the total length of the trajectory. The square root of the sum of the squared differences in each coordinate gives the distance between two points in 3D space. This feature was calculated individually for the dominant and nondominant eyes, resulting in two distinct features of this type.	2

### Statistical analysis for performance evaluation

Extracted features—comprising 84 search information features, 84 temporal network flexibility features, and 12 eye-tracking features—were used as independent variables to develop random intercept models for evaluating performance. The random intercept model accounts for the within-subject variability. The goal was to find the features that are associated with performance among different subjects. Seven-fold cross-validation was used to reduce individual effects in detecting important features (i.e., predictors). Forward feature selection was used to identify the possible predictors. Variables selected at least twice during cross-validation were considered as possible predictors. These potential predictors were then used to develop the final linear random intercept models for performance evaluation. To quantify the variation in the output variable explained by the independent variables in the model, Efron’s pseudo-R-square was computed. Mean Absolute Error (MAE), and Root Mean Square Error (RMSE) metrics were computed to assess the performance evaluation models’ performance.

### Statistical analysis for learning rate evaluation

In our analysis, all features extracted from EEG and eye-tracking data were considered continuous variables. Linear regression was used to analyze the learning rate, a suitable method given that each subject exhibits a unique learning rate for each task. Eye-tracking and EEG features from the first attempt, along with baseline performance scores and age, were used as potential factors in a multivariate linear regression analysis to identify the most significant factors (i.e., features). Subjects with high initial performance scores typically exhibit lower learning rates. For example, a subject scoring 95 out of 100 is less likely to achieve a steep learning rate compared to one who scores 60. Therefore, we use the first-attempt performance score as a baseline in analyzing learning rates. We considered the initial performance score as a baseline to adjust for individual variances among subjects. Forward feature selection was used to identify the predictors of learning rate. The identified features were used to develop the learning rate evaluation model. To assess how well the independent variables explain the variance in the dependent variable (learning rate), the R^2^ metric was calculated. MAE, and RMSE metrics were calculated to assess the learning rate evaluation models’ performance.

#### Regression models’ terms

In the regression models, the term ‘estimate’ reflects the variation in the outcome variable (e.g., performance score) for a one-standard deviation shift in the predictor variable. The standard error of an estimate indicates the standard deviation of its sampling distribution.

### Relationship between hours of experience with RAS and performance

We employed Pearson correlation analysis to investigate the relationship between hours of experience with RAS and performance.

### Relationship between performance and mental workload

It has been frequently reported that performance and mental workload mutually influence each other^95^. We employed Pearson correlation analysis to investigate the relationship between the two factors in this study.

All tests were two-sided with a level of significance set at 0.05. Statistical analyses were conducted using SAS® (version 9.4, SAS Institute Inc., Cary, NC, USA).

### Reporting summary

Further information on research design is available in the Nature Research Reporting Summary linked to this article.

### Supplementary information

Reporting summary

Supplementary Tables

## Data Availability

The data analyzed in the current study are available at Shafiei et al.^96^. 10.13026/9m3f-ac20.

## Code availability

No custom code or mathematical algorithm was developed for this study. Details regarding the specific codes used can be found in the references cited. Any access restrictions or licensing information associated with the codes used can be obtained from the respective sources as indicated in the references.
