# Supplementary material for: Development of performance and learning rate evaluation models in robot-assisted surgery using electroencephalography and eye-tracking
Source: NPJ Sci Learn. 2024 Jan 20;9:3. doi: 10.1038/s41539-024-00216-y (PMC10799032; doi:10.1038/s41539-024-00216-y)
Supplement: Supplementary file 1 — Reporting summary [file 41539_2024_216_MOESM1_ESM.pdf]

Corresponding author(s): Somayeh B Shafiei

Last updated by author(s): Jan 3, 2024

Reporting Summary

Nature Portfolio wishes to improve the reproducibility of the work that we publish. This form provides structure for consistency and transparency in reporting. For further information on Nature Portfolio policies, see our Editorial Policies and the Editorial Policy Checklist.

Statistics

For all statistical analyses, confirm that the following items are present in the figure legend, table legend, main text, or Methods section.

n/a	Confirmed	
☐	☒	The exact sample size (*n*) for each experimental group/condition, given as a discrete number and unit of measurement
☒	☐	A statement on whether measurements were taken from distinct samples or whether the same sample was measured repeatedly
☒	☐	The statistical test(s) used AND whether they are one- or two-sided<br>*Only common tests should be described solely by name; describe more complex techniques in the Methods section.*
☐	☒	A description of all covariates tested
☐	☒	A description of any assumptions or corrections, such as tests of normality and adjustment for multiple comparisons
☐	☒	A full description of the statistical parameters including central tendency (e.g. means) or other basic estimates (e.g. regression coefficient) AND variation (e.g. standard deviation) or associated estimates of uncertainty (e.g. confidence intervals)
☒	☐	For null hypothesis testing, the test statistic (e.g. *F*, *t*, *r*) with confidence intervals, effect sizes, degrees of freedom and *P* value noted<br>*Give P values as exact values whenever suitable.*
☒	☐	For Bayesian analysis, information on the choice of priors and Markov chain Monte Carlo settings
☒	☐	For hierarchical and complex designs, identification of the appropriate level for tests and full reporting of outcomes
☒	☐	Estimates of effect sizes (e.g. Cohen's *d*, Pearson's *r*), indicating how they were calculated

Our web collection on statistics for biologists contains articles on many of the points above.

Software and code

Policy information about availability of computer code

Data collection	The EEGO software from ANT Neuro was purchased and used for EEG data recording.
Data analysis	The advanced source analysis (ASA) framework from Ant Neuro was purchased and used for EEG data pre-processing. The MATLAB R2020b was purchased and used for EEG data analyses.

For manuscripts utilizing custom algorithms or software that are central to the research but not yet described in published literature, software must be made available to editors and reviewers. We strongly encourage code deposition in a community repository (e.g. GitHub). See the Nature Portfolio guidelines for submitting code & software for further information.

Data

Policy information about availability of data

All manuscripts must include a data availability statement. This statement should provide the following information, where applicable:

- Accession codes, unique identifiers, or web links for publicly available datasets
- A description of any restrictions on data availability
- For clinical datasets or third party data, please ensure that the statement adheres to our policy

The data analyzed in the current study are available at: Shafiei, S. B., Shadpour, S., Mohler, J., Seilanian Toussi, M., Doherty, P., & Jing, Z. (2023). Electroencephalogram and eye-gaze datasets for robot-assisted surgery performance evaluation (version 1.0.0). PhysioNet.
This dataset was published by Physionet. https://doi.org/10.13026/9m3f-ac20

Research involving human participants, their data, or biological material

Policy information about studies with human participants or human data. See also policy information about sex, gender (identity/presentation), and sexual orientation and race, ethnicity and racism.

Reporting on sex and gender	The study design did not consider the variables of sex and gender. Sex and/or gender information was obtained through self-reporting, and in all cases, the reported sex and gender matched. However, the study did not conduct a sex/gender-based analysis. The primary objective of this study was to develop models for evaluating surgical performance and learning rate using EEG and eye-tracking data. The significant disparity in sample sizes between males and females may introduce biases. As a result, the study did not consider sex/gender as a factor in the analysis.
Reporting on race, ethnicity, or other socially relevant groupings	Race, ethnicity, or other socially relevant groupings were not considered in the manuscript.
Population characteristics	Age was considered in analysis. Average age of participants was 35.5 years and standard deviation was 11.7 years.
Recruitment	Employees at our institute were invited to the study through an invitation email or verbal invitation. There was no self-selection or other biases that may impact results.
Ethics oversight	Roswell Park Comprehensive Cancer Center's Institutional Review Board (IRB: I-241913)

Note that full information on the approval of the study protocol must also be provided in the manuscript.

Field-specific reporting

Please select the one below that is the best fit for your research. If you are not sure, read the appropriate sections before making your selection.

☒ Life sciences ☐ Behavioural & social sciences ☐ Ecological, evolutionary & environmental sciences

For a reference copy of the document with all sections, see nature.com/documents/nr-reporting-summary-flat.pdf

Life sciences study design

All studies must disclose on these points even when the disclosure is negative.

Sample size	Power analyses was done to determine sample size.
Data exclusions	The Local Outlier Factor (LOF) algorithm was used to detect outlier samples and exclude those from the analyses.
Replication	All information for reproducing findings is available in the manuscript (Methods).
Randomization	This study was not a randomized control study.
Blinding	This study was not a randomized control study.

Reporting for specific materials, systems and methods

We require information from authors about some types of materials, experimental systems and methods used in many studies. Here, indicate whether each material, system or method listed is relevant to your study. If you are not sure if a list item applies to your research, read the appropriate section before selecting a response.

Materials & experimental systems

n/a	Involved in the study	
☒	☐	Antibodies
☒	☐	Eukaryotic cell lines
☒	☐	Palaeontology and archaeology
☒	☐	Animals and other organisms
☒	☐	Clinical data
☒	☐	Dual use research of concern
☒	☐	Plants

Methods

n/a	Involved in the study	
☒	☐	ChIP-seq
☒	☐	Flow cytometry
☒	☐	MRI-based neuroimaging

Plants

Seed stocks	N.A.
Novel plant genotypes	N.A.
Authentication	N.A.
