# Supplementary material for: Development of performance and learning rate evaluation models in robot-assisted surgery using electroencephalography and eye-tracking
Source: NPJ Sci Learn. 2024 Jan 20;9:3. doi: 10.1038/s41539-024-00216-y (PMC10799032; doi:10.1038/s41539-024-00216-y)
Supplement: Supplementary file 2 — Supplementary Tables [file 41539_2024_216_MOESM2_ESM.pdf]

Supplementary Information

Supplementary Table 1 Results of a linear random intercept model for performance evaluation at the Tubes task without subject-wise standardization

Predictors	Estimate	p-value
Average pupil diameter of dominant eye	-0.72	0.01
Average temporal flexibility in BA 18 at beta-band frequencies	0.54	0.02
number of samples =61; subject was not a significant random effect (p-value=0.10); pseudo R^2=0.54		

Supplementary Table 2 Results of a linear regression model for learning rate evaluation at the Tubes task without subject-wise standardization

Predictors	Estimate	p-value
Performance at the first attempt	-0.35	$5x10^{-4}$
Average temporal flexibility in BA 46 at alpha-band frequencies	-0.87	$7x10^{-4}$
Average temporal flexibility in BA 18 at theta-band frequencies	-0.59	0.03
number of samples = 26; R^2= 0.64		

Supplementary Table 3 Results of a linear random intercept model for performance evaluation at the Suture Sponge task without subject-wise standardization

Predictors	Estimate	p-value
Average temporal flexibility in BA 10 at beta-band frequencies	0.55	0.003
Average search information in BA 5 at theta-band frequencies	1.07	0.002
number of samples = 66; subject was a significant random effect (p-value=0.01); pseudo R^2=0.75		

Supplementary Table 4 Results of a linear regression model for learning rate evaluation at the Suture Sponge task without subject-wise standardization

Predictors	Estimate	p-value
Average temporal flexibility in BA 45 at theta-band frequencies	-0.27	0.003
Average search information in BA 45 at theta-band frequencies	-1.03	< 0.001
Average search information in BA 41 at gamma-band frequencies	1.002	0.01
number of samples = 26; R^2= 0.71		

Supplementary Table 5 Results of a linear random intercept model for performance evaluation at the Dots and Needles task without subject-wise standardization

Predictors	Estimate	p-value
Average search information in BA 45 at beta-band frequencies	-1.12	0.01
Average temporal flexibility in BA 45 at alpha-band frequencies	0.24	0.02
number of samples = 66; subject was a significant random effect (p-value=0.01); pseudo R^2=0.71		

Supplementary Table 6 Results of a linear regression model for learning rate evaluation at the Dots and Needles task without subject-wise standardization

Predictors	Estimate	p-value
Average temporal flexibility in BA 37 at alpha-band frequencies	0.67	0.03
Average temporal flexibility in BA 39 at theta-band frequencies	0.62	0.01
Average search information in BA 9 at alpha-band frequencies	1.09	< 0.001
number of samples = 26; R^2=0.60		
